# Brain MR findings in patients treated with particle therapy for skull base tumors

**DOI:** 10.1186/s13244-019-0784-9

**Published:** 2019-09-23

**Authors:** Gisela Viselner, Lisa Farina, Federica Lucev, Elena Turpini, Luca Lungarotti, Ana Bacila, Alberto Iannalfi, Emma D’Ippolito, Barbara Vischioni, Sara Ronchi, Enrico Marchioni, Francesca Valvo, Stefano Bastianello, Lorenzo Preda

**Affiliations:** 1 Diagnostic Imaging Unit, National Center of Oncological Hadrontherapy (CNAO), 27100 Pavia, Italy; 2 Neuroradiology Department, IRCCS Mondino Foundation, Pavia, Italy; 3 Diagnostic Radiology Residency School, University of Pavia, Pavia, Italy; 4 Department of Diagnostic and Interventional Radiology and Neuroradiology, Fondazione IRCCS Policlinico San Matteo, Pavia, Italy; 5 Radiotherapy Unit, National Center of Oncological Hadrontherapy (CNAO), Pavia, Italy; 6 Unit of Neuro-oncology, IRCCS Mondino Foundation, Pavia, Italy; 7 Department of Brain and Behavioral Sciences, University of Pavia, Pavia, Italy; 8 Department of Clinical, Surgical, Diagnostic and Pediatric Sciences, University of Pavia, Pavia, Italy

**Keywords:** Hadrontherapy, Radiation necrosis brain, Multiparametric magnetic resonance imaging

## Abstract

Nowadays, hadrontherapy is increasingly used for the treatment of various tumors, in particular of those resistant to conventional radiotherapy. Proton and carbon ions are characterized by physical and biological features that allow a high radiation dose to tumors, minimizing irradiation to adjacent normal tissues. For this reason, radioresistant tumors and tumors located near highly radiosensitive critical organs, such as skull base tumors, represent the best target for this kind of therapy. However, also hadrontherapy can be associated with radiation adverse effects, generally referred as acute, early-delayed and late-delayed. Among late-delayed effects, the most severe form of injury is radiation necrosis. There are various underlying mechanisms involved in the development of radiation necrosis, as well as different clinical presentations requiring specific treatments. In most cases, radiation necrosis presents as a single focal lesion, but it can be multifocal and involve a single or multiple lobes simulating brain metastasis, or it can also involve both cerebral hemispheres. In every case, radiation necrosis results always related to the extension of radiation delivery field. Multiple MRI techniques, including diffusion, perfusion imaging, and spectroscopy, are important tools for the radiologist to formulate the correct diagnosis. The aim of this paper is to illustrate the possible different radiologic patterns of radiation necrosis that can be observed in different MRI techniques in patients treated with hadrontherapy for tumors involving the skull base. The images of exemplary cases of radiation necrosis are also presented.

## Key points

- Hadrontherapy provides a more beneficial dose distribution to tumors than conventional radiotherapy.
- Among late-delayed adverse effects, radiation necrosis is the most severe one.
- Clinical presentation of radiation-related injury is often asymptomatic or slightly symptomatic.
- Late-delayed focal lesions are typically contrast-enhancing areas with necrotic core.
- Radiation-related brain lesions generally show high signal intensity on ADC maps.

## Introduction

Since the discovery of X-rays by Röntgen in 1895, photon and electron beams have been widely used in the management of malignant tumors as a conventional radiotherapy (RT) and they currently play a crucial role in the treatment of brain and skull base tumors, either used alone or as adjuvant therapy, combined with surgery and chemotherapy [1].

On the other hand, since 1946, when Wilson R. firstly proposed the medical use of proton for cancer therapy, interest in particle beams expanded and the efficacy of heavy ions for clinical use had been largely investigated. Nowadays, particle beams with protons or carbon ions have been gradually applied in clinics.

In comparison with conventional RT, ion beams such as protons and carbon ions provide a more beneficial dose distribution, as they have different physical characteristics. While the conventional radiation generally passes through a biological target with an equivalent dose delivered throughout all the beam path, the particle beams release energy at the inverse of their velocity.

As a result, photons are characterized by an exponential absorption of dose with depth while protons and heavy ions—such as carbon ions—are featured by an inverted depth dose profile: energy deposition increases with penetration depth, reaching the maximum, known as the Bragg peak, at the end of their path.

As the original Bragg peak is too narrow and sharp to completely cover the target lesion, a broadening of the narrow peak (spread-out Bragg peak or SOBP) according to the size of the lesion is used in cancer treatment.

This results in particle beams allowing a highly localized deposition of energy that can be utilized for increasing radiation dose to tumors while minimizing irradiation to adjacent normal tissues.

Thus, the integral dose is low and the treatment is always extremely conformal to the target volume, although there are some differences between proton beams and carbon ion beams: in the region beyond the distal end of the peak, almost no dose is deposited with protons, while a small dose is deposited with carbon ions [2].

Regarding biological aspects, protons release a larger mean energy per unit length (linear energy transfer or LET) than photons, but their radiobiologic properties do not differ substantially from those of photons: these low-LET radiations commonly cause single-strand DNA break but some cancer cells have mechanisms for repairing this kind of damage and may survive after treatment [2, 3].

Carbon ion beams, which are high-LET radiations, not only have the favorable physical properties of protons but also have a superior biologic advantage in comparison with protons or photons: they commonly cause double-strand DNA break by one hit, the most significant event for cancer cell death, reducing the possibility for the tumor cells to repair radiation injury [2, 4].

High-LET heavy ions show further biological advantages compared with photons: a greater number of hypoxic cells (which are usually radioresistant to conventional radiotherapy and may form a large part of the tumor) are killed and there is less variation in cell cycle-related radiosensitivity.

Finally, Tsujii and Kamada reported a potential suppression of metastases, probably related to double-strand DNA breaks [2–7].

In conclusion, protons and carbon ions have either the physical advantage of better spatial selectivity or a higher radiobiological efficacy than that of photons, or both and as a result, the ideal indication for using hadrontherapy is the treatment of radioresistant tumors or tumors located near highly radiosensitive critical organs, such as skull base tumors [4].

Among these, clival chordomas, chondrosarcomas, sinonasal malignancies, adenoid cystic carcinomas with perineural spread are currently treated with hadrontherapy.

However, even if hadrontherapy has the advantage to allow a high dose to the tumor, minimizing the dose delivered to healthy surrounding tissues, in the treatment of intracranic, skull base, and sinonasal cancers, certain parts of central nervous system might be included in the radiation area, resulting to be at risk for radiation damage [1, 8]. In particular, in treatment of sinonasal malignancies, anterior-inferior frontal lobes are known to be at risk, as well as brain stem and cerebellum in treatment of skull base malignancies and temporal lobes in nasopharyngeal carcinomas and clival chordomas and chondrosarcomas [9, 10].

It is well known that adverse effects associated with radiation therapies, both conventional and using particle beams, are generally referred to as acute, early-delayed and late-delayed.

Acute and early-delayed radiation effects occur within the first 3 months after treatment and typically spontaneously resolve, while late-delayed radiation effects may occur from few months to several years after treatment and are often progressive [1, 8, 11, 12].

Late radiation injury is one of the most serious complications of RT for head-and-neck tumors. The spectrum of late radiation injury ranges from faint damage to white matter to complete ischemic necrosis [8, 13].

Among late-delayed effects, the most severe form of injury is radiation necrosis (RN), which was first described in 1930 by Fischer and Holfelder [14, 15].

Radiological features of brain radiation-induced damage in acute, early-delayed, and late-delayed phases after hadrontherapy for skull base tumors, observed using conventional and advanced magnetic resonance imaging (MRI) techniques, are presented in this paper.

## Pathophysiology

The underlying mechanisms leading to the development of brain radiation necrosis are still current subject of study. However, several factors which may contribute to the development of radiation-induced central nervous system (CNS) toxicity have been described, as injury of white matter glial cells and particularly oligodendrocyte damage, vessel damage, and alterations of cytokine expression.

Among neural cells, the most affected are oligodendrocytes and their precursors, which are responsible for the formation of myelin sheaths, thus causing subsequent demyelination and astrocytic gliosis, while a relative sparing of gray matter is observed. The supposed mechanism involved is a direct damage [16, 17].

It is well known that alterations of the vasculature are crucial for the development of CNS alterations in response to irradiation. Radiation causes injury to endothelial cells resulting in vessel wall necrosis, vascular ectasia, and telangiectasia, which lead to an increased permeability and to the disruption of the blood–brain barrier, with a consequent vascular disorder and vasogenic edema [18]. Progressive vascular changes lead to thrombosis and infarction, followed by necrosis of the surrounding parenchyma [14].

Recent studies have shown the crucial role of various cytokines, which amplify the cascade of events [19–24]. Endothelial cells of cerebral vessels are initially damaged by irradiation, resulting in tissue hypoxia. In surrounding tissues, reactive astrocytes, microglia, and macrophages respond to hypoxia expressing hypoxia-inducible factor (HIF)-1α. HIF-1α strongly mediates upregulation of vascular endothelial growth factor (VEGF). VEGF has two important biological properties: one is a strong angiogenic peptide component, which may produce telangiectasis as pathological angiogenesis, and the other one is a vascular permeability factor causing an increase of perilesional vasogenic edema [19–21].

Consequently to blood-brain barrier disruption, inflammatory cells are able to cross into the extravascular space where they secrete many cytokines such as tumor necrosis factor (TNF)-α, which is proved to be responsible of the increased vascular permeability and thus of the vasogenic edema [22–24].

### Incidence

The reported incidence in literature of radiation necrosis related to particle beam therapy is very variable and in some studies proton and carbon ion RT also show significantly different incidence curves [6, 8, 25–27].

Miyawaki et al. analyzed the incidence of radiation-induced brain injury after protons and carbon ions in patients with head and neck and skull base tumors who had received partial RT to the brain. Of 59 patients analyzed, 48 patients were treated with proton therapy and 11 patients with carbon ion RT: eight (17%) patients treated with proton therapy and seven (64%) patients treated with carbon ion therapy developed MRI findings of radiation-induced brain injury [8].

Santoni et al. analyzed the incidence of temporal lobe injury after proton therapy. Of 96 patients analyzed, ten (10.4%) developed clinical symptoms and MRI changes consistent with radiation necrosis [28].

Schlampp et al. reported that ten (6%) of 59 patients treated with carbon ion RT for chordomas and low-grade chondrosarcomas of the skull base developed radiation-induced temporal lobe reactions on MRI [25].

Regarding to the detection of radiation injury, the time interval between treatment and detection associated with carbon ion therapy is longer than the one observed with proton or photon therapy.

Miyawaki et al. reported mean latency time between treatment and brain injury onset ranges between 6–49 months for proton therapy and 11–41 months for carbon ion therapy [8].

Schlampp et al. reported median latency time until appearance of the first temporal lobe injury of 14 months for carbon ions [25].

It has been reported that tissue damage after carbon ion therapy appears more frequently, is more severe, and lasts longer than after proton therapy; this seems due to the higher relative biological efficacy (RBE) of carbon ions compared to protons [29].

### Clinical presentation and treatment

The clinical presentation of the radiation-related injury may vary from being nearly asymptomatic to focal or diffuse neurological deficits; patients may present with increased intracranial pressure due to edema, cognitive dysfunction, seizures, or neurological deficits related to the position of the lesion or, as observed in the majority of the cases, may be nearly asymptomatic or slightly symptomatic.

Neurological symptoms of presentation are commonly classified using the European Organization for Research and Treatment of Cancer (EORTC), Radiation Therapy Oncology Group (RTOG), or National Cancer Institute’s Common Terminology Criteria for Adverse Events (CTCAE) scales [6, 8, 25, 30, 31].

Asymptomatic patients with radiation necrosis may only require close surveillance and no specific treatment, while symptomatic patients with radiation necrosis are commonly treated with corticosteroids, which inhibit the pro-inflammatory response associated with the injury and thus the edema [32]. In some rare severe cases, surgery is required to relieve mass effect secondary to the lesion and to the edema [11].

New specific treatments, which interfere with the underlying mechanisms perpetuating the damage, have been proposed. Vascular endothelial growth factor (VEGF) has recently been reported to play an important role in the development of radiation necrosis. In this regard, bevacizumab, a monoclonal anti-VEGF antibody, has been reported to be effective in treating radiation necrosis and reducing perilesional edema [33–36].

Kishimoto et al. reported a better recovery from radiation injury after carbon ion therapy than after conventional photon therapy, probably due to the relatively small irradiated volume of normal brain as a consequence of the excellent dose-localizing properties of particle beam therapy [13].

Evidence of brain injury is usually obtained by follow-up observations and image findings, especially those from magnetic resonance imaging (MRI).

Therefore, there is a growing interest in using imaging techniques, either structural or functional, to assist in this hard diagnosis.

## Imaging features of hadrontherapy-related brain injury

### Magnetic resonance imaging protocol

MRI, supported by most advanced techniques (such as diffusion, perfusion imaging, and magnetic resonance spectroscopy), due to its high sensitivity and its capacity to provide detailed information of brain structures, represents the basic survey for diagnosis and follow-up in head and neck tumors [1].

MRI protocols in diagnostic examination and in post-treatment follow-up must include at least one fluid sensitive sequence (such as turbo spin echo, TSE T2 or fluid attenuated inversion recovery, FLAIR), even though a multiplanar assessment of the region is advisable; a spin echo (SE) T1 sequence should be performed as well. Furthermore, given the information that can derive from the calculation of apparent diffusion coefficient (ADC) values, diffusion-weighted imaging (DWI) sequences are now routinely performed. After contrast agent administration, isotropic, three-dimensional 3D GE T1 FS sequences guarantee an optimal multiplanar view.

Magnetic resonance spectroscopy (MRS) and perfusion-weighted imaging (PWI) are included into protocol study if requested by the physician when abnormal brain findings are detected during basic MRI investigation.

### Conventional MRI sequences

Radiation damage can be divided according to onset timing in acute, early-delayed and late-delayed, each may have specific imaging characteristics, also related to physiopathogenetic mechanism on their basis.

Acute injury typically occurs during or shortly after the radiation treatment [31]. MRI findings of acute brain damage consist of high signal on fluid sensitive sequences due to vasogenic edema involving the white matter, which is related to increased vessel permeability resulting from the damage of the endothelium of capillaries and arterioles. As no blood-barrier breakdown is observed in this stage, no focal contrast enhancement is shown on T1 images. Acute alterations usually regress spontaneously [9].

Early-delayed injury, which can be found within few weeks after treatment ending, consists in transient demyelination associated with altered permeability of vessels and it results into a hyperintense alteration of white matter on T2/FLAIR imaging. The gray matter adjacent to the altered white matter can be swollen with a slightly increased signal on T2/FLAIR imaging, with loss of distinction between gray and white matter. Frequently contrast enhancing areas after gadolinium administration are recognized due to alterations of the blood-brain barrier [9]. Although this kind of damage can be spontaneously reversible, it may require differential diagnosis with recurrence or secondary localization [31].

Late delayed radiation brain injury can occur within a few months to several years after treatment completion, and its spectrum ranges from slight damage to white matter to complete ischemic necrosis. Subcortical arcuate fibers are usually spared, although lesions occasionally extend to the adjacent cortex or deep gray matter [14]. Radiation necrosis is an irreversible and often progressive process [12, 31].

Although in most cases late injury presents as a single focal lesion, it is not uncommon to find multifocal brain injury, which can involve a single or even multiple lobes and simulate brain metastasis, however always related to the extension of radiation delivery field. Moreover, cases of involvement of both cerebral hemispheres can also be found.

Late delayed focal lesions typically appear at MRI as contrast-enhancing areas with central necrotic core. Enhancing solid portions of the lesion are iso-hypointense on T2-weighted sequences, while necrotic core has high T2 signal; usually, the lesion is surrounded by a widespread hyperintensity on T2/FLAIR due to vasogenic edema.

Nowadays, there are no purely radiological classifications addressing the severity of late damage, also because radiological findings do not always correlate with clinical ones. In particular, it is possible to experience cases of symptomatic patients in the presence of faint alterations but also cases of asymptomatic patients with extensive necrotic alterations.

Late Effects Normal Tissue Task Force subjective, objective, management and analytic (LENT-SOMA) scoring system, a multifactorial classification that also takes into account radiological aspects of lesions integrated with objective and subjective semeiotics, was proposed in 1995 by the EORTC and the RTOG.

**Fig. 1:**
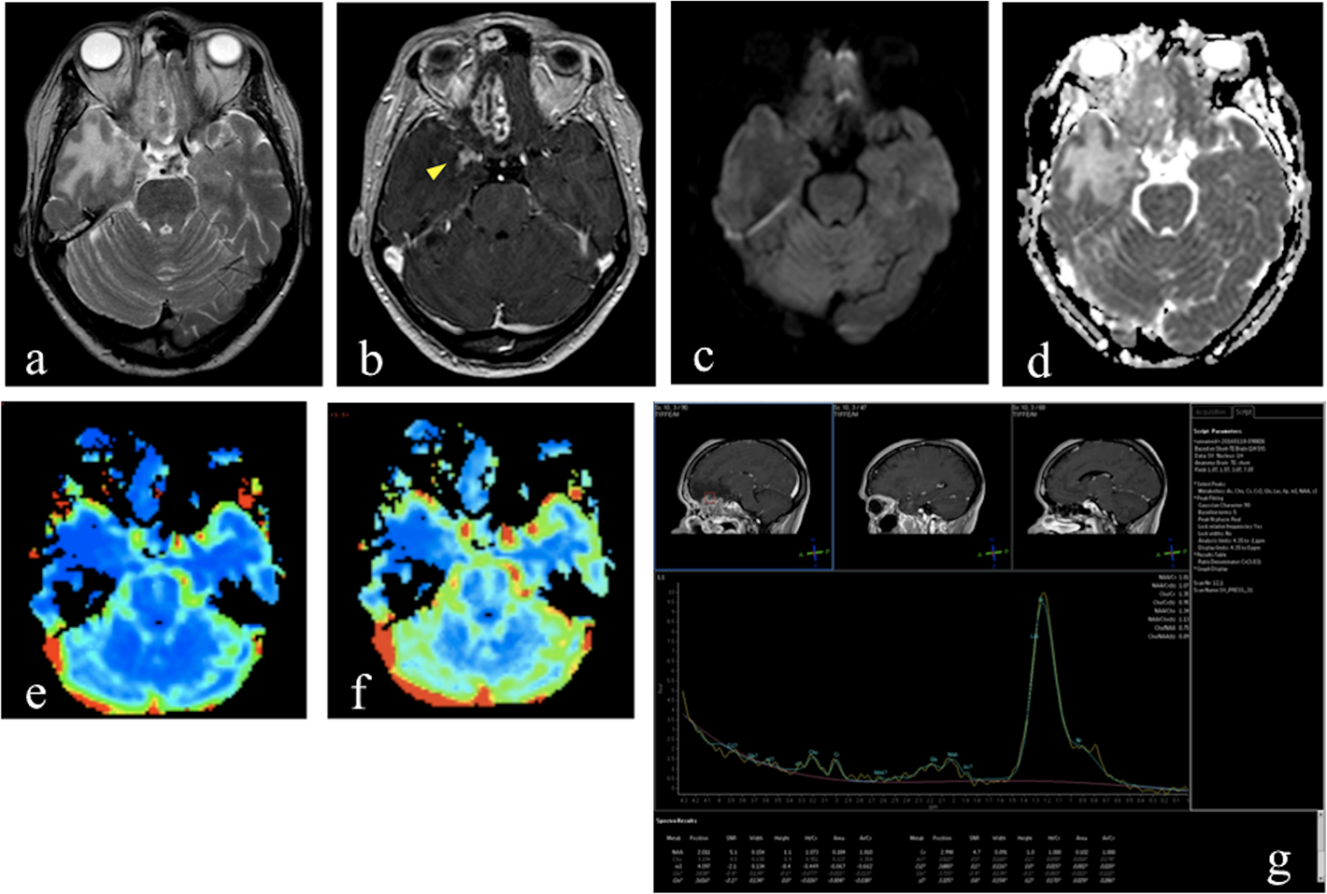
MRI performed 16 months after carbon ion therapy of a sino-nasal adenoid-cystic carcinoma. T2-weighted image (**a**) shows an extensive area of altered signal intensity involving right gyrus rectus, polar and mesial temporal regions. Axial postcontrast T1-weighted image (**b**) shows two different areas of enhancement: a focal area of enhancement without necrosis, surrounded by edema, as a grade 1 injury of Late Effects Normal Tissue Task Force subjective, objective, management and analytic (LENT-SOMA) scoring system in right mesial temporal region (yellow arrowhead); a larger area with non-enhancing portion of necrotic tissue with peripheral rim enhancement, surrounded by edema, in right gyrus rectus. The axial diffusion weighted (DWI) image (**c**) and apparent diffusion coefficient (ADC) map (**d**) show no diffusion restriction within the enhancing area with an evident hyperintense area in ADC map. Cerebral blood flow (CBF) (**e**) and relative cerebral blood volume (rCBV) (**f**) maps show no hyperperfusion in the enhancing area. Magnetic resonance spectroscopy (MRS)-short TE (**g**) reveals the presence of high lipid and lactate peaks, which is typical of radiation necrosis

**Fig. 2:**
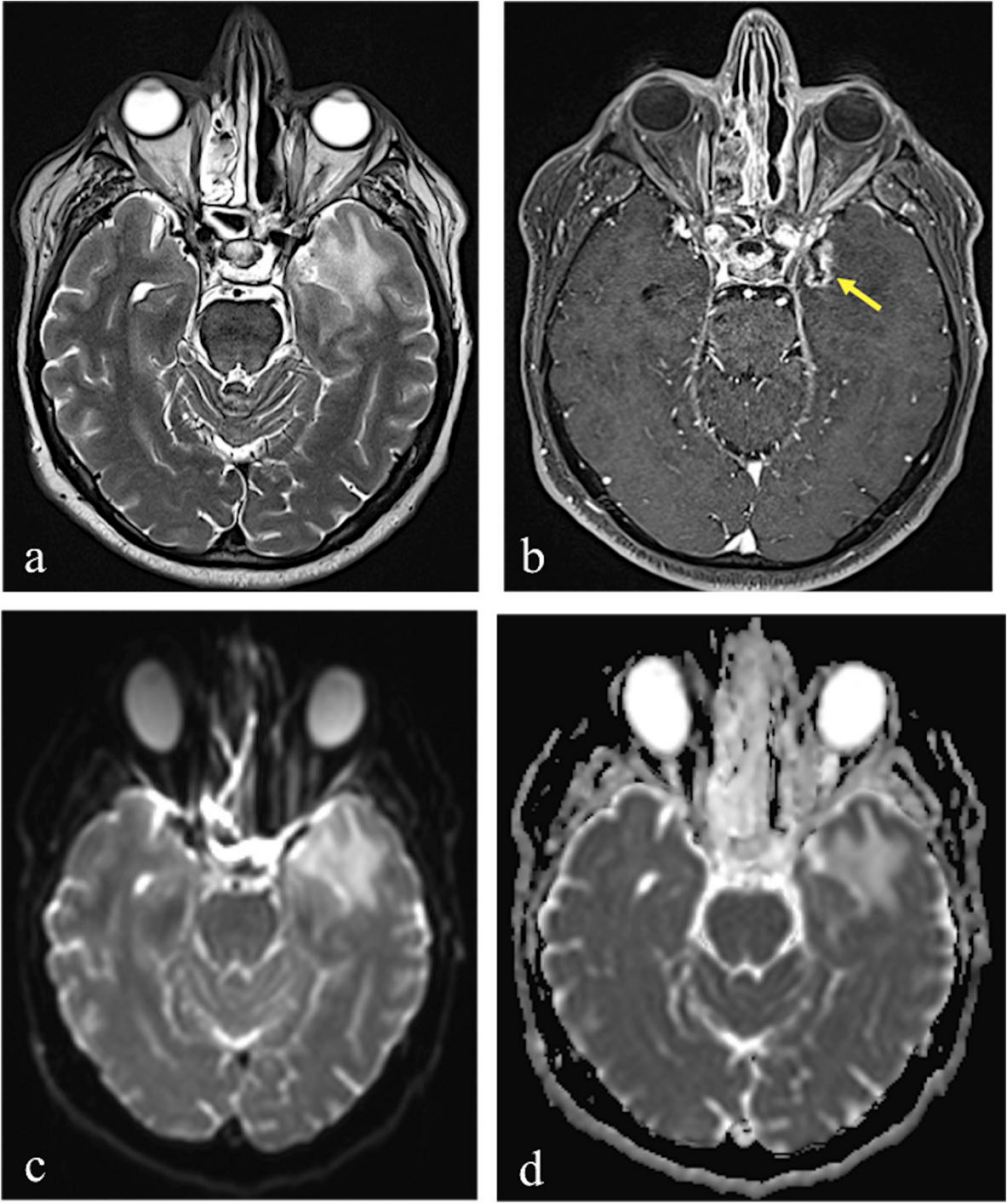
MRI performed 20 months after 68.8 Gy carbon ion therapy in a patient who previously underwent surgical resection for sinonasal adenoid cystic carcinoma. Axial T2-weighted image (**a**) shows hyperintensy involving subcortical white matter and gray matter in left polar and mesial temporal regions. Axial post-contrast T1-weighted image (**b**) reveals the presence of a well-defined contrast-enhancing lesion with central necrotic core (yellow arrow), as in grade 2 injury of LENT-SOMA scoring system. DWI shows no diffusion restriction within the enhancing area, with an hyperintense signal in both *DWI*-*b1000* (**c**) and ADC map (**d**), because of “T2-shine through” effect

**Fig. 3:**
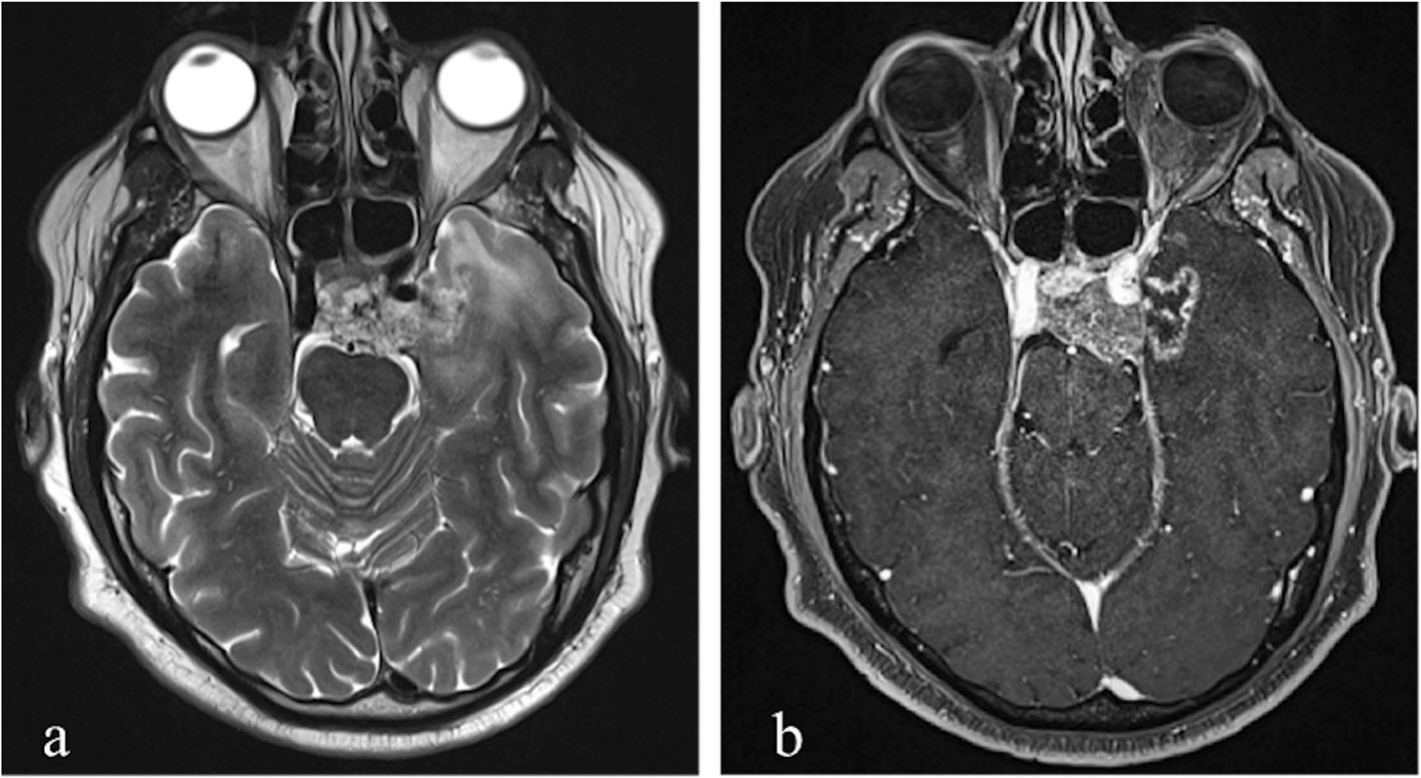
MRI performed 14 months after 74 Gy Proton-beam therapy in a patient who previously underwent subtotal surgical resection of clival chordoma. Axial T2-weighted image (**a**) shows hyperintensity involving subcortical white matter in left polar temporal region right adjacent to clival structures. Axial post-contrast T1-weighted image (**b**) reveals a focal non-enhancing portion related to necrotic tissue with a peripheral rim enhancement, as in grade 2 injury of LENT-SOMA scoring system

**Fig. 4:**
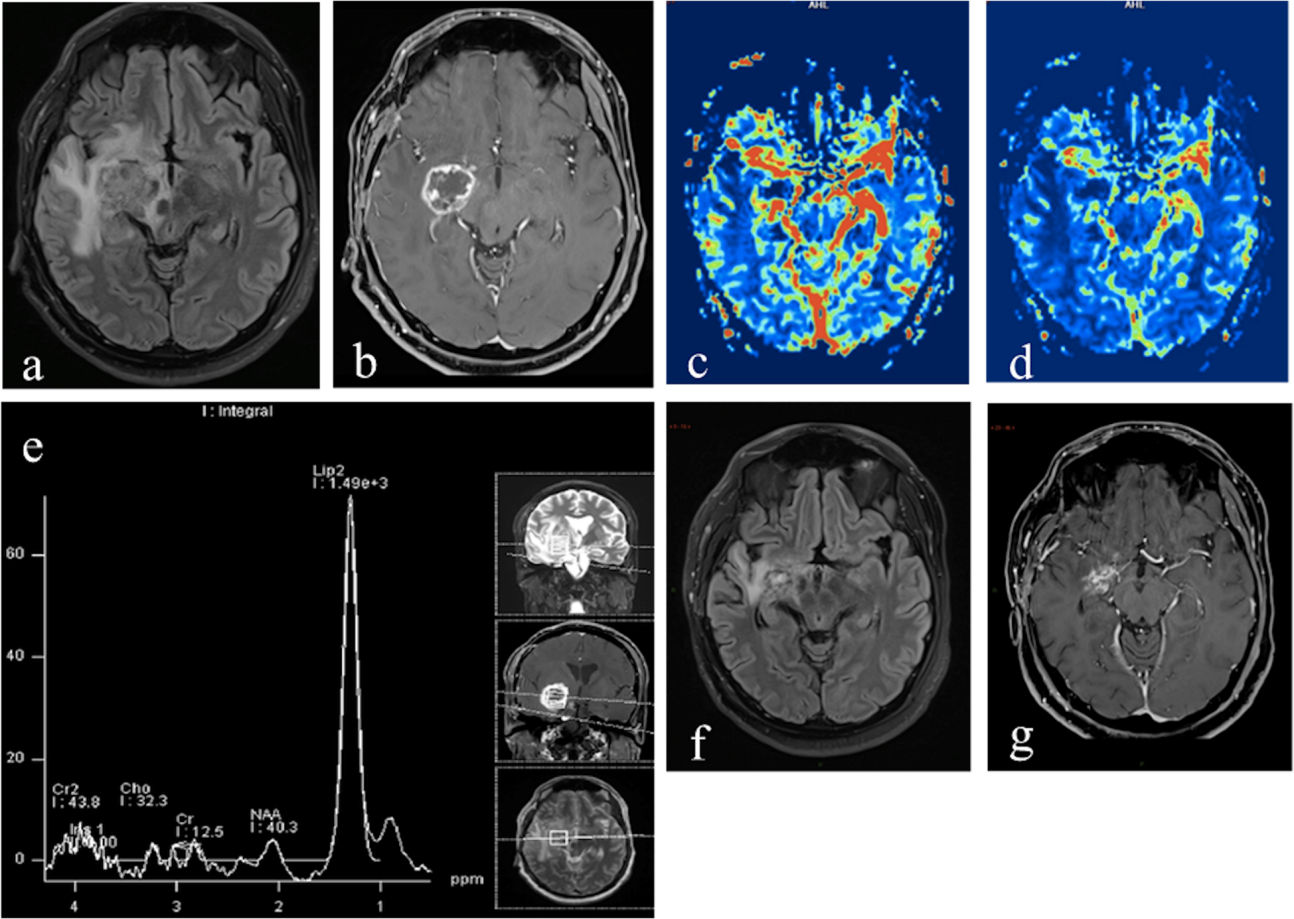
MRI performed 13 months after proton therapy for clival chondrosarcoma. Axial fluid-attenuated inversion recovery (FLAIR) image (**a**) shows a disomogeneous area of altered signal, with hypo- and hyperintense components, located in the right hippocampal region and surrounded by extensive edema involving subcortical temporal and frontoinsular white matter, the cortico-spinal tract and causing mass effect on the right cerebral peduncle. Axial postcontrast T1-weighted image (**b**) shows peripheral and disomogenous enhancement, with “soap bubble” like pattern. rCBV (**c**) and CBF (**d**) maps show no hyperperfusion in the enhancing area. MRS-short TE (**e**) reveals the presence of high lipid and lactate peak with general decrease of other metabolites, highly suggestive for radiation necrosis. Control MRI performed in the same patient after 3 months; no intercurrent therapies. Axial FLAIR image (**f**) shows an evident decrease of the vasogenic edema, confined to temporal lobe. Axial T1-post contrast-weighted image (**g**) shows reduction in size of the enhancing lesion

**Fig. 5:**
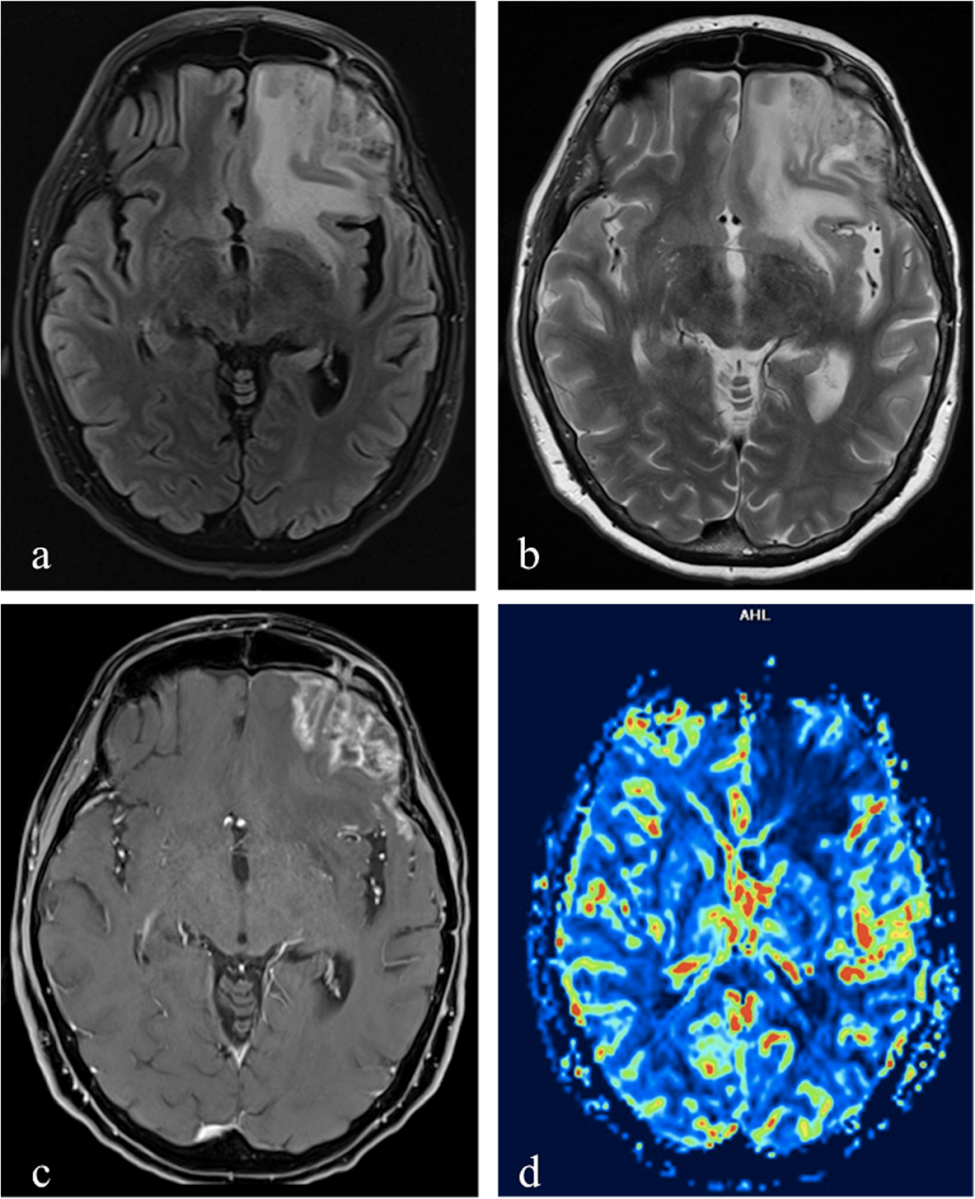
MRI performed 5 years after proton-beam therapy for skull base chondrosarcoma. Axial FLAIR (**a**) and T2-weighted (**b**) images show a disomogenous hyperintense lesion, involving both gray and white matter, in left frontal lobe surrounded by wide edematous area extending to the external capsula and causing mild mass-effect on striatum nucleus and midline structures. Axial post-contrast T1-weighted image (**c**) shows disomogeneous enhancement with “Swiss cheese” pattern. rCBV map (**d**) demonstrates no hypervascularization within the enhanced area

In LENT-SOMA scoring system [37], MRI findings can be graded as follows:

- Grade 1. Change in focal white matter, focal contrast enhancement, and surrounding edema; owing to blood-brain barrier damage, after gadolinium administration focal enhancement is observed in T1 sequences, surrounded by an hyperintense alteration on T2/FLAIR due to edema. No areas of focal necrosis are seen in grade 1 injury (Fig. 1a, b).
- Grade 2. Non-enhanced area or a cyst in the enhanced lesion; in this stage in T1 after gadolinium administration a focal non-enhancing portion related to necrotic tissue can be seen inside the enhancing solid area, giving a characteristic appearance of rim enhancement (Figs. 2a, b and 3). Grade 2 and higher injuries can be referred as radiation necrosis [31]. A focal T2-hyperintense cystic lesion can also be found to replace the necrotic tissue in a later phase [14].
- Grade 3. Focal necrosis leading to mass effect. As the lesion increases in size, a local mass effect is typical, which can be either direct or due to extensive vasogenic edema. In the literature, particular models of radiation necrosis have been described. "Soap-bubble" pattern refers to lesions involving white matter with peculiar irregularly-enhancing necrotic core; "Swiss cheese" pattern refers to a diffuse involvement of white matter and cortex interspersed with necrotic foci (Figs. 4a, b and 5a–c).
- Grade 4. Uncommon finding, characterized by large cystic lesions hypointense on T1-weighted images and hyperintense on T2/FLAIR; faint contrast enhancement can be seen on cyst wall and peripheral hyperintense T2 signal involving white matter is constantly assessed [10]. As they can reach remarkable size, they can develop severe mass effect representing life-threatening complication and require surgical intervention.

**Fig. 6:**
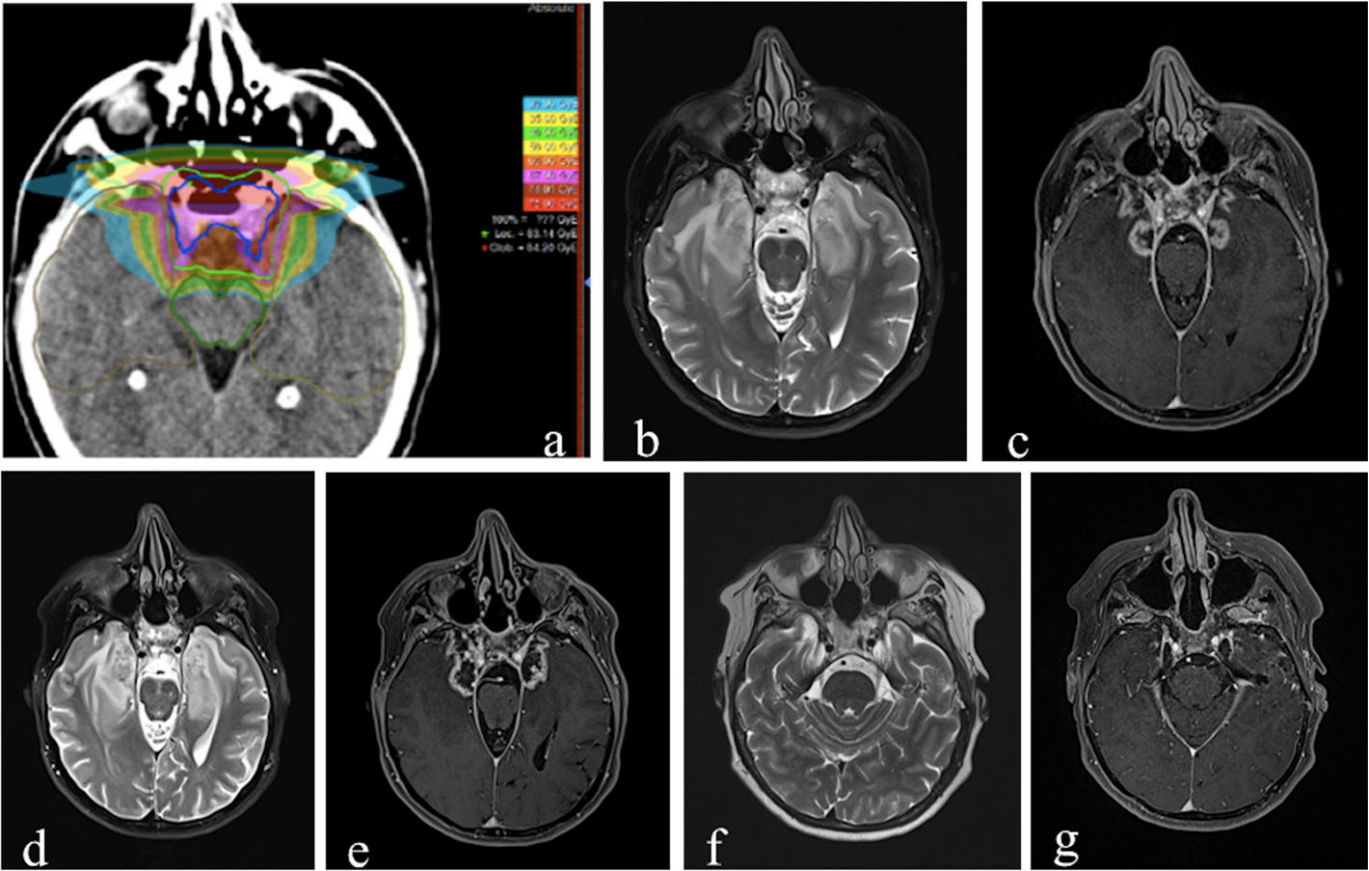
MRI performed in a patient who underwent 74 Gy proton-beam therapy for skull base chordoma. Radiation treatment field (**a**). After 18 months from the end of the treatment, axial T2-weighted image (**b**) shows diffuse hyperintensity involving predominantly white matter in temporal lobes and axial postcontrast T1-weighted image (**c**) demonstrates irregular-shaped enhancing areas in both mesial and polar temporal regions. The morphology of the enhancing lesions shown is in accordance with the radiation treatment field shown in (**a**). At the following follow-up, performed 21 months after the end of the treatment, axial T2-weighted image (**d**) and axial postcontrast T1-weighted image (**e**) show that the lesions and the surrounding edema shown in (**b**) and (**c**) have increased in volume. The morphology of the enhancing lesions shown remains in accordance with the radiation treatment field shown in (**a**). After 48 months from the end of the treatment, T2-weighted image (**f**) and axial T1 post-contrast-weighted image (**g**) show the evolution of the alterations, demonstrating focal atrophic changes in both temporal poles according with the evident decrease of extension of the contrast enhancing lesions

As radiation necrotic lesions progress, they can lead to severe shrinkage of cortex and, especially, of the white matter, resulting in focal brain atrophy [14, 38] (Fig. 6).

### Diffusion-weighted imaging

Diffusion-weighted imaging (DWI) is a functional MRI technique which uses the random motion of water molecules due to their thermal energy inside a medium, also known as diffusion motion or “Brownian motion,” to acquire images. In tissues, water molecules do not freely diffuse and their movement occurs largely in the extracellular space where it is affected by several factors, such as the structure of intra- and extracellular space, viscosity of the medium, and the degree of cellularity [39–41].

DW images are typically acquired using strong magnetic field gradients to make the magnetic resonance signal sensitive to the molecular motion of water.

The diffusion-sensitizing effect from the gradients is indicated by the *b* value (in seconds per square millimeter), which is defined by the gradient strength, the duration of the gradients, and the time interval between the gradients; DWI sequences are routinely acquired using different *b* values.

By examining the signal intensity on DW images obtained with different *b* values, the amount and speed of movement of water molecules can be estimated and quantified by using ADC, which represents direction independent water displacement [39, 42].

In tissues with high cellularity, free water motion is restricted and there is a high signal intensity on high *b* value DW images with low signal intensity on the ADC map; whereas in tissues with low cellularity, as in case of apoptosis or necrosis, the signal intensity is low on high *b* value DW images, while it is high in the corresponding ADC maps.

Lesions of radiation-induced brain injury may have a variety of signal patterns on DW images and ADC maps and this is explained by the coexistence of several pathophysiological processes in the same lesion, such as liquefactive necrosis, ischemic necrosis, reactive vasogenic edema, gliosis, or fibrosis and inflammatory infiltrate [43].

Commonly brain lesions induced by radiation therapy can be visualized either as high or sometimes low signal intensity areas on high *b* value DW images, but generally show high signal intensity on the corresponding ADC map [38, 39, 44, 45] (Fig. 1c, d).

When a lesion is hyperintense on both the DWI image and the corresponding ADC map, the phenomenon is referred to as T2 *shine*-*through* effect [40] (Fig. 2c, d).

**Fig. 7:**
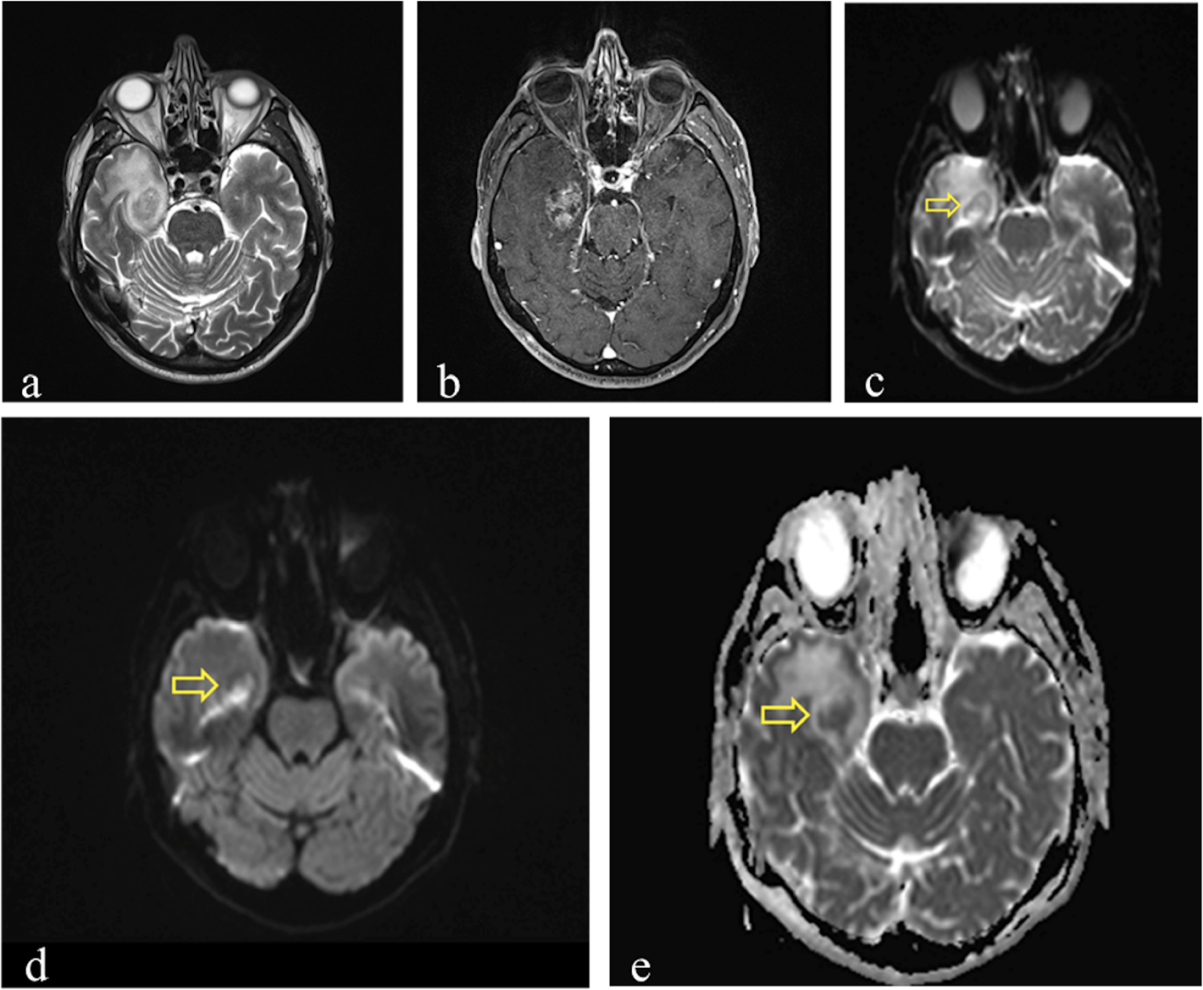
MRI performed 18 months after 65.6 Gy carbon ion therapy in a patient with sinonasal adenoid cystic carcinoma. Axial T2-weighted image (**a**) shows a subcortical white matter and gray matter lesion in right polar and mesial temporal regions. Axial post contrast T1-weighted image (**b**) shows the presence of inhomogeneous contrast-enhancing lesion. Diffusion imaging documented the presence of foci of restricted diffusion within the medial part of the lesion appearing as hyperintense areas in DWI-*b0* (**c**) and DWI-*b1000* images (**d**) and hypointense on the corresponding ADC map (**e**) (yellow arrows)

In some cases, it is possible to document radiation necrosis lesions with high signal intensity on high *b* value DWI and low ADC values, which is often evident in the enhancing portion of the lesions, and this is probably due to the high viscosity and cellular composition of the inflammatory infiltrate in early necrosis or it can be secondary to the scarring from gliosis or fibrosis within the lesion in later phases [14, 43, 46] (Fig. 7).

Similarly also hemorrhagic foci, which can be present within areas of necrosis, may decrease ADC values probably because of hypercellularity or hyperviscosity [47].

### Perfusion-weighted imaging

Perfusion refers to microcirculation blood supply of a tissue and perfusion imaging techniques allow quantitative evaluation of tissutal microcirculation by capturing the first pass of intravenously administrated paramagnetic contrast agent.

Perfusion-weighted imaging (PWI) techniques can offer hemodynamic parameters useful in surveying radiation injury and, if necessary, in distinguishing radiation injury from tumor recurrence, the latter being characterized by an increased perfusion related to neoangiogenesis.

The most widespread perfusion methods are two: dynamic susceptibility-weighted contrast enhanced (DSC MR imaging) and dynamic contrast enhanced (DCE MR imaging).

DSC is the most widely used perfusion technique for brain because its utilization has many advantages: better signal-to-noise ratio (SNR), shorter scan times and greater availability; nevertheless, its application can be limited due to susceptibility artifacts from hemorrhage, surgical material and intraparenchimal calcifications [1].

It is an advanced MR technique that exploits the property of a high flow injected gadolinium bolus to induce a loss of signal in the T2-weighted sequences to evaluate a capillary blood volume perfusion. Several parameters can be extrapolated from it, such as the relative cerebral blood volume (rCBV), the relative peak height (rPH), and the percentage of signal recovery (PSR) [9].

rCBV is an indirect index of cerebral blood volume, as it provides information about blood perfusion of a region of interest (ROI) related to its healthy counterpart.

Since radiation necrosis is associated with regions of reduced perfusion due to vascular endothelial damage and coagulative necrosis induced by treatment [1], and vascular lesions in radionecrosis involve a combination of broad fibrinoid necrosis and dilatation of blood vessels [48], rCBV is expected to be decreased in late radiation injury [49]. (Figs. 1e, f, 4c, d and 5d).

A study aiming to determine the value of perfusion-sensitive contrast-enhanced MRI for differentiating tumor recurrence from non-neoplastic enhancing tissue in post-treatment intra-axial brain cancer, performed on 20 patients who developed new enhancing lesions, suggested a threshold of normalized rCBV ratio lower than 0.6 as highly indicative for radiation injury [50].

**Fig. 8:**
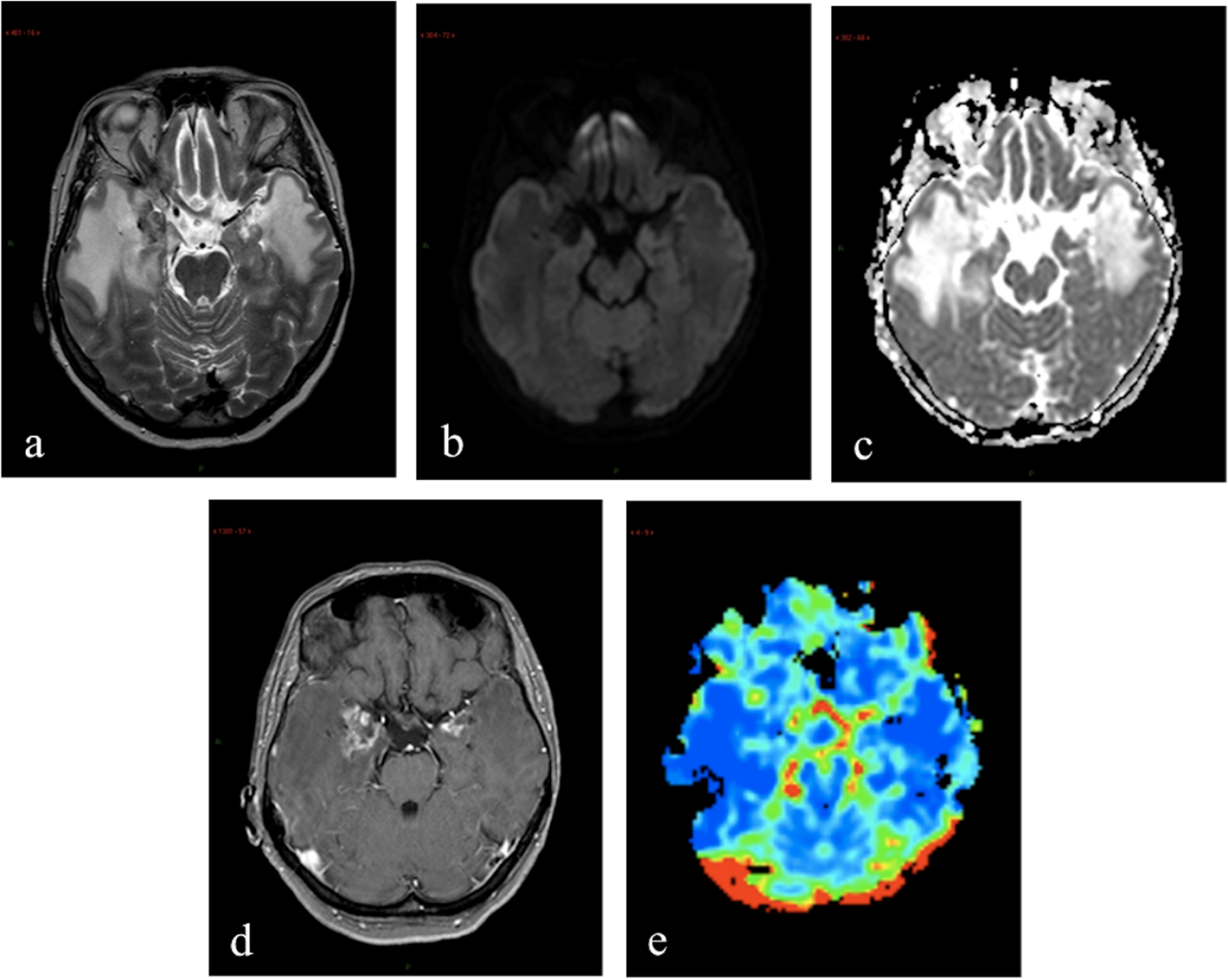
MRI performed 22 months after 78.8 Gy carbon ion therapy in a Patient with malignant peripheral nerve sheath tumor. T2-weighted image (**a**) shows disomogeneous areas of altered signal, with hypo- and hyperintense components, located in mesial temporal regions both on the right and on the left, and surrounded by extensive edema involving subcortical polar and mesial temporal white matter. DWI documents no diffusion restriction within the enhancing areas with evident hyperintense areas in ADC map on both sides (**b, c**). Axial post contrast T1-weighted image (**d**) shows the presence of inhomogeneous contrast-enhancing lesions in mesial temporal regions both on the right and on the left. rCBV map (**e**) shows hyperperfusion of the left enhancing area and no hyperperfusion of the enhancing area on the right

Actually, treatment effects on vascular bed which lead to radiation necrosis also include intermediate steps of non-obstructive inflammatory damage (capillary aneurysmal dilatation and elongation, teleangectasias) resulting in an increased blood flow, especially in enhancing areas of the lesion; as rCBV is commonly calculated on a ROI placed on the entire enhancing lesion, increased perfusion can lead to higher rCBV values, even on normal white matter (Fig. 8) [14, 41].

rPH represents the maximum change in signal intensity as contrast agent passes through capillary vascularization; peak values are first calculated by the difference in one region of interest before and after contrast administration, then obtaining a ratio with those of the healthy counterpart [51].

rPH has been proven to be highly correlated to rCBV [52, 53] and seems to have low values in radiation necrosis.

Studies conducted on patients treated for glioblastoma multiforme to evaluate any differences in these hemodynamic parameters between groups with histologically proven tumor recurrences and others with radiation necrosis, showed how mean, minimum, and maximum values of rPH and rCBV were significantly higher in tumor recurrence; rPH values higher than 2.17 were observed only in recurrent tumors [54].

Tissutal vascular permeability can be measured by PSR, which is an additional parameter that provides information about contrast agent leakage through capillary circle, while higher rPSR values are expected in radiation necrosis compared to recurrent tumors, in literature is reported a large degree of rPSR values overlapping between the two [54]; this aspect makes the PSR a less reliable parameter in the characterization of radiation damage.

In DCE MRI, rapidly acquired T1-weighted sequences are performed to detect changes in signal during the passage of contrast bolus through vessels, which can be influenced from overall perfusion, microvascularization permeability, and extracellular volume.

DCE-MRI is based on a two-compartmental (plasma space and extravascular-extracellular space) pharmacokinetic model. The simplest and widely used model is the Kety/Tofts one, which makes it possible to estimate *K*^trans^ (volume transfer constant between blood plasma and extravascular extracellular space), *v*_e_ (volume of the extravascular extracellular space per unit volume of tissue), and *k*_ep_ (rate constant between extravascular extracellular space and blood plasma, where *k*_ep_ = *K*^trans^/*v*_e_) [55, 56].

*K*^trans^, the most measured DCE parameter, can have different interpretations depending on blood flow and permeability. When there is very high permeability, the flux of gadolinium-based contrast agent is limited only by flow, and thus *K*^trans^ mainly reflects blood flow. In conditions of very low permeability, the gadolinium-based contrast agent cannot leak easily into the extravascular-extracellular space, and thus *K*^trans^ mainly reflects permeability. Despite this complexity, *K*^trans^ appears to reproducibily measure permeability in glioma patients [56] and DCE-MRI is used mainly as a tool to measure the permeability of the capillary wall. Because of the vascular pauperization in radiation injury lesions and consequently the small volume of gadolinium accumulating in extravascular space in affected brain areas, low *K*^trans^ values are expected [57, 58] while higher *K*^trans^ values are expected in tumor recurrence. However, the radiation-induced endothelial damage could lead to an increased capillary permeability in radiation necrosis with feedback of *K*^trans^ values higher than expected [59].

In comparison to DSC, some advantages have been reported for DCE, such as higher spatial resolution and no susceptibility to artifacts. Nevertheless, the main hindrance with DCE is represented by the non-linear relationship between T1 signal intensity and contrast agent concentration; even if pharmacokinetic models (such as Tofts-Kermode model) have been developed to outflank this issue, DCE remains incline to errors in quantifying hemodynamic parameters.

In particular, in order to differentiate tumor recurrence and radiation necrosis in treated high-grade gliomas, Zakhari et al. [59] reported that DSC-derived CBV measurement is more accurate compared with DCE-derived parameters including the permeability parameter (*K*^trans^). They also reported that a combination of T1 and T2 perfusion parameters showed no significant improvement in diagnostic accuracy compared with CBV.

In a retrospective review of 40 patients using DSC and DCE, Seeger et al. [60] reported better diagnostic performance for DSC in distinguishing recurrent high-grade gliomas from stable disease.

For all these evidences, the utilization of DCE in common practice is limited.

### Magnetic resonance spectroscopy

Magnetic resonance spectroscopy (MRS) measures the relative compositions of various metabolites in brain tissue, most commonly including *N*-acetylaspartate (NAA), choline (Cho), creatine (Cr), lipid, and lactate [1].

NAA represents neuronal density and it decreases in case of neuronal injury or death; creatine level is the result of cellularity and cellular metabolism; choline is representative of cellular turnover and proliferative activity since it is a metabolite found in cell membrane biosynthesis; lipids and lactate are the products of anaerobic metabolism and cell destruction, therefore they are not normally present in brain tissue [61].

Radiation-induced lesions may show a variety of MRS findings, changing over time, which range from only a mild decrease in NAA to very low or undetectable levels of all metabolites [62].

In particular, the NAA decrease which occurs early after treatment has been suggested to be related either to neuronal damage, such as neuronal cell death due to apoptosis, or to neuronal dysfunction secondary to the irradiation [63].

**Fig. 9:**
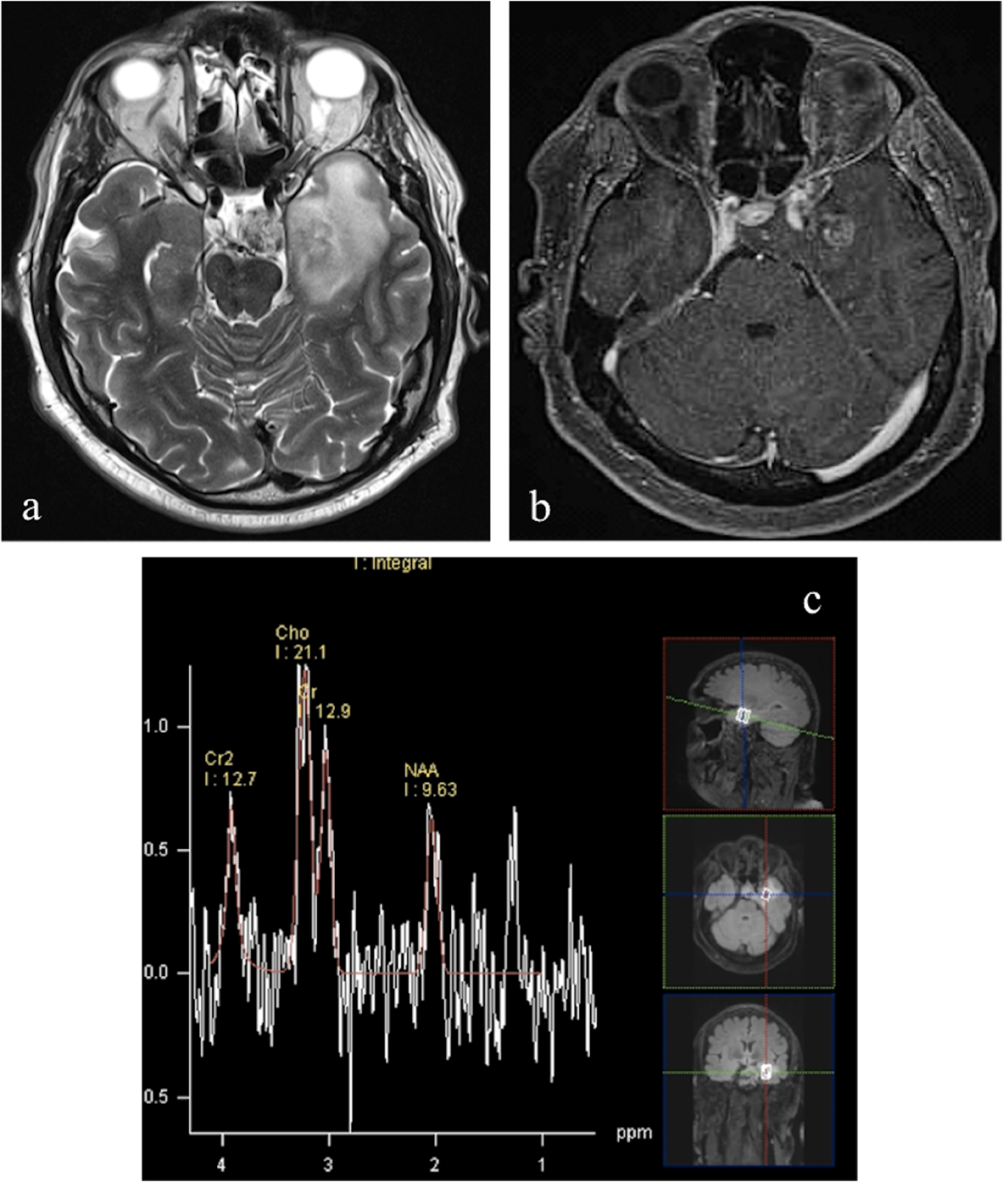
MRI performed 10 months after 74 Gy proton-beam therapy in the same patient of Fig. 3, who previously underwent subtotal surgical resection of clival chordoma. Axial T2-weighted image (**a**) shows hyperintensy involving subcortical white matter and gray matter in left polar and mesial temporal regions right adjacent to clival structures. Axial post-contrast T1-weighted image (**b**) reveals the presence of a round-shaped contrast-enhancing lesion within the hyperintense area. Multi-voxel MRS (**c**) shows a choline (Cho) peak and an increase/inversion of choline/N-acetylaspartate (Cho/NAA) ratio, which may reflect inflammatory cells infiltrate in early brain damage

**Fig. 10:**
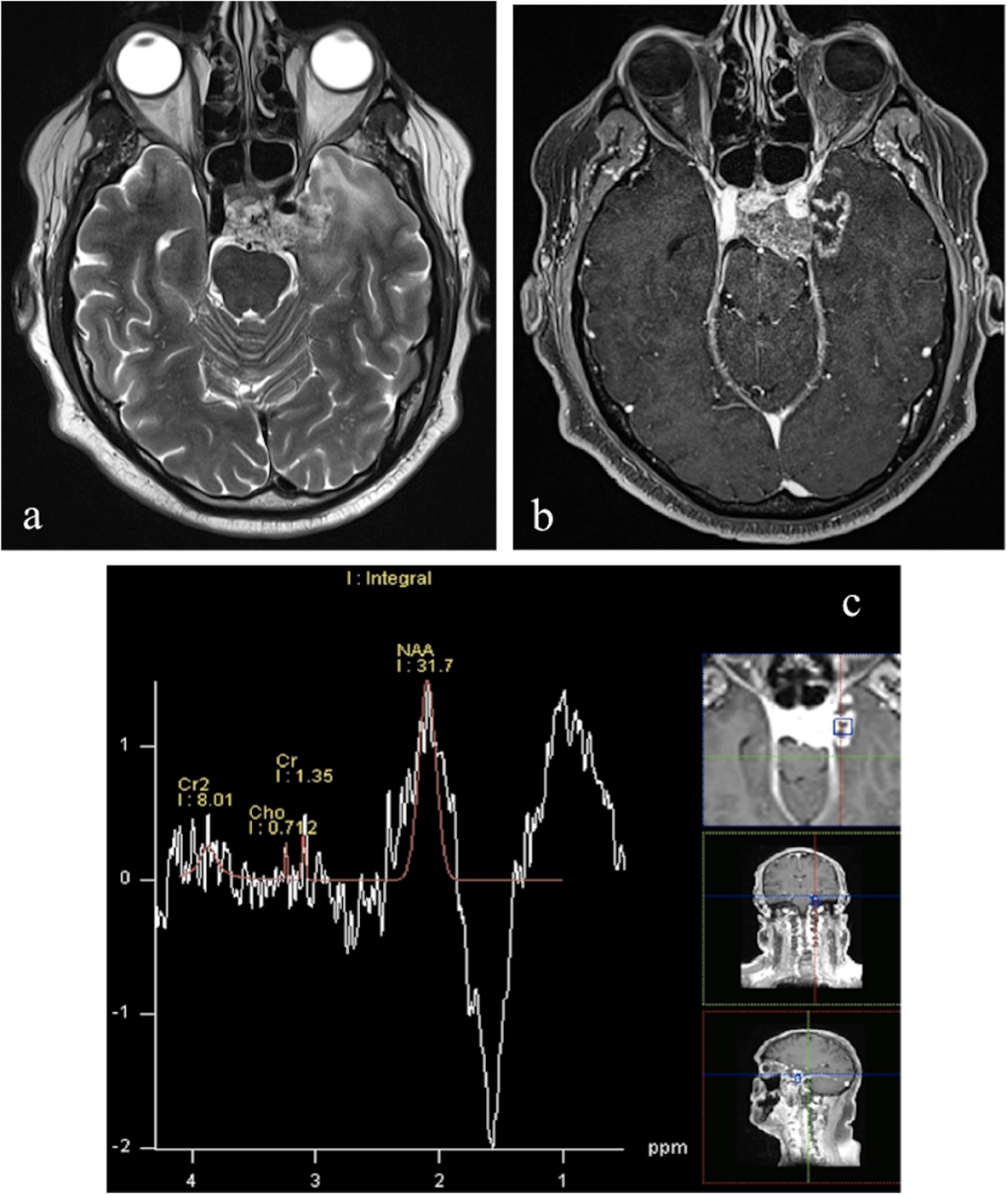
MRI performed in the same patient as in Figs. 3 and 9, 14 months after proton-therapy. Axial T2-weighted image (**a**) shows decrease of extension of the hyperintense area in left temporal region. Axial post-contrast T1-weighted image (**b**) reveals the presence of a better defined contrast enhancing lesion. Multi-voxel MRS with long TE (**c**) performed after 4 months shows evident decrease in Choline peak and the appearance of a lactate-lipid peak, typical for radionecrosis

Radiation necrosis has been reported to determine variable changes in choline and creatine levels [62–65]. For example, choline may increase in the first few weeks of radiation treatment due to its release from damaged cell membranes and the presence of inflammatory cells (Fig. 9), and subsequently decrease to levels below those of normal tissue as treatment necrosis appears [1, 63, 65–67] (Fig. 10).

As a result, some studies report a transitory inversion of Cho/NAA ratio which may reflect brain injury occurring early after radiation treatment [68, 69].

Observations of a decrease in both the Cho and the Cho compounds as well as a decrease in the Cho/Creatine (Cr) ratio have also been reported in irradiated brain [70, 71].

However, the typical spectrum in delayed cerebral necrosis shows a generalized decrease of Cho, Cr, and NAA [51].

Lactate and lipid peaks start to manifest at approximately week 4 after radiation therapy and continue to increase during the delayed phase of radiation changes, likely as a consequence of the liberation of intracellular lipid bodies and of cell membrane damage due to massive cellular destruction [9] (Figs. 1g and 4e).

A more recent study on tissue-based, untargeted metabolomics analysis to examine differential metabolic profiles of radiation necrosis in comparison to tumoral tissue, identified multiple candidate metabolites which could assist in the diagnosis. For example, alpha-tocopherol and citric acid were most significantly elevated in radiation necrosis compared with tumor, while proline and UDP-glucuronic acid were most elevated in tumoral tissue compared with radiation necrosis [72].

## Conclusions

In conclusion, hadrontherapy is increasingly used for the treatment of various tumors resistant to conventional radiotherapy or located near highly radiosensitive critical organs. In particular, skull base tumors such as clival chordomas, chondrosarcomas, sinonasal malignancies, and adenoid cystic carcinomas with perineural spread are currently treated with hadrontherapy.

Radiation can be associated with adverse effects generally referred to as acute, early-delayed, and late-delayed. Among late-delayed effects, the most severe form of injury is radiation necrosis.

Brain radiation-induced necrosis can present with heterogeneous radiological features and with different patterns. Multiple MRI techniques including diffusion, perfusion imaging, and spectroscopy play an important role in the evaluation and diagnosis of brain radiation necrosis. Radiologist should be aware of the typical imaging manifestations of brain radiation necrosis, which are described in this paper.

## Data Availability

Data sharing is not applicable to this article as no datasets were generated or analyzed during the current study.

## Abbreviations

ADC: Apparent diffusion coefficient
Cho: Choline
CNS: Central nervous system
Cr: Creatine
CTCAE: National Cancer Institute’s Common Terminology Criteria for Adverse Events
DCE: Dynamic contrast enhanced magnetic resonance imaging
DSC-MRI: Dynamic susceptibility-weighted contrast enhanced magnetic resonance imaging
DWI: Diffusion-weighted imaging
EORTC: European Organization for Research and Treatment of Cancer
FLAIR: Fluid attenuated inversion recovery
HIF-1α: Hypoxia-inducible factor
LENT-SOMA: Late Effects Normal Tissue Task Force subjective, objective, management and analytic
LET: Linear energy transfer
MRI: Magnetic resonance imaging
MRS: Magnetic resonance spectroscopy
NAA: *N*-acetylaspartate
PSR: Percentage of signal recovery
PWI: Perfusion-weighted imaging
rCBV: Relative cerebral blood volume
RN: Radiation necrosis
ROI: Region of interest
rPH: Relative peak height
RT: Radiotherapy
RTOG: Radiation Therapy Oncology Group
SE: Spin echo
SNR: Signal-to-noise ratio
SOBP: Spread-out Bragg peak
TNF-α: Tumor necrosis factor
TSE: Turbo spin echo
VEGF: Vascular endothelial growth factor
